# Delafloxacin Resistance and MIC-Distribution in Ciprofloxacin-Resistant Enterobacterales: A Retrospective Evaluation of Minimum Inhibitory Concentrations

**DOI:** 10.3390/antibiotics15090934

**Published:** 2026-09-20

**Authors:** Patrick Forstner, Isabel Klugherz, Hanna Greimel, Kathrin Jäntsch, Ute Wagner-Eibel, Melanie Palz, Laura Krieger, Ivo Steinmetz, Karl Dichtl

**Affiliations:** 1 Diagnostic and Research Institute of Hygiene, Microbiology and Environmental Medicine, Medical University of Graz, 8010 Graz, Austria; patrick.forstner@medunigraz.at (P.F.);; 2 Institute of Laboratory Medicine and Microbiology, Protestant Hospital of Bethel Foundation, Campus Bielefeld-Bethel, University Hospital OWL of Bielefeld University, 33617 Bielefeld, Germany

**Keywords:** delafloxacin, ciprofloxacin, epidemiology, surveillance, antibiotic susceptibility testing, antibiotic resistance

## Abstract

**Objectives:** The fourth-generation fluoroquinolone delafloxacin (DFX) is propagated as a novel (oral) treatment option for infections with multidrug-resistant Gram-negatives. To date, efficacy data for Enterobacterales is only available for wild-type isolates but not for those with pre-existing resistance against third-generation fluoroquinolones. **Methods:** One hundred twenty-nine non-duplicate clinical isolates of Enterobacterales that demonstrated ciprofloxacin resistance in VITEK 2 testing were collected and analyzed for CIP and DFX minimum inhibitory concentrations (MICs) via broth microdilution. **Results:** DFX MICs ranged from 0.5 to ≥ 16 mg L^−1^ (median: 4 mg L^−1^). All *Escherichia coli* isolates (*n* = 56; 43% of all isolates), the only species with EUCAST breakpoints, were classified as DFX resistant. All other isolates belonging to species for which EUCAST epidemiological cut-offs (ECOFFs) are available (*n* = 64; 50% of all isolates) demonstrated MICs above the ECOFF. With a minimum and median MIC of 2 and 8 mg L^−1^, respectively, the remaining isolates (6%) achieved similar results. A moderate correlation between the MIC values of the two fluoroquinolones was found. For the majority of isolates (64%), other oral options for antibiotic therapy, such as amoxicillin/clavulanic acid and trimethoprim/sulfamethoxazole, were still available. **Conclusions:** Our data suggests that, compared to CIP, DFX does not demonstrate increased in vitro efficacy against fluoroquinolone-resistant Enterobacterales. Subsequent DFX testing seems to be of limited additional value.

## 1. Introduction

Fluoroquinolones are a class of bactericidal, broad-spectrum antibiotics targeting bacterial topoisomerases [1,2]. These enzymes catalyze and reseal DNA cleavage in order to interconvert relaxed and supercoiled forms of DNA [1,2,3]. Nalidixic acid, the first quinolone, was found in 1962 as a by-product of the synthesis of the antimalarial drug chloroquine [4]. In the following decades, numerous synthetic drugs derived from nalidixic acid were developed and later classified on the basis of their in vitro activity. Whereas first-generation quinolones were only active against aerobic, Gram-negative bacteria, and substances of the second-generation, including ciprofloxacin (CIP) and levofloxacin (LVX), added antimicrobial activity against aerobic Gram-positive bacteria while improving activity against Gram-negative bacteria [4,5]. Third-generation fluoroquinolones later increased potency against Gram-positive bacteria and added anaerobic coverage [4,5]. The subsequent introduction of fourth-generation fluoroquinolones further increased the activity against anaerobes and Gram-positive organisms [4,5]. Resistance against most fluoroquinolones is acquired by a single-point mutation in a highly conserved region in one of the genes encoding subunits of the polymeric topoisomerases, e.g., gyrA or parC [6,7].

Currently, CIP and LVX represent essential components of our therapeutic repertoire for treating infections affecting various organ systems [1,2]. Their oral bioavailability and capability to effectively penetrate tissue is particularly advantageous [1,2,8]. As a result, the World Health Organization (WHO) lists fluoroquinolones as one of five critically important antimicrobials in human medicine [9].

However, frequent use and, presumably, misuse have led to increasing resistance rates to CIP and LVX, raising concern about the effectiveness of fluoroquinolones in empirical anti-infective therapy [2]. Delafloxacin (DFX), classified as a fourth-generation fluoroquinolone, might be a valuable addition to our antimicrobial repertoire, combining the advantages of an established drug class with the benefits of a novel substance. In 2017 and 2019, respectively, DFX was approved in the USA and the European Union (EU) for the treatment of acute bacterial skin and skin structure infections (ABSSSI). The authorization has since been extended to include community-acquired pneumonia (CAP) in both the USA and EU. The anionic structure of this new compound confers increased efficacy under acidic conditions, such as those present in infected tissues [10,11]. Available data indicates good efficacy against Gram-positive and Gram-negative pathogens, including multi-resistant pathogens such as methicillin-resistant *Staphylococcus aureus* (MRSA) and carbapenem-resistant organisms (CRO) [12,13,14], which makes them promising options in clinical practice [8,13,14]. Additionally, DFX, like most fluoroquinolones, also shows excellent bioavailability when taken orally [8].

As knowledge about resistance epidemiology is the prerequisite for the empirical use of an antimicrobial substance, acquisition of this data is of utmost importance. Although information on DFX resistance has increased recently, these investigations focus on specific clinical conditions, e.g., arthritis or keratitis [6,15], or specific bacterial genera, e.g., *Corynebacterium* or *Ureaplasma* [16,17]. However, data on Enterobacterales is still scarce, as there are only a few publications available, and all of those were published more than a decade ago [18,19]. In light of the emergence and spread of carbapenem-resistant Enterobacterales, which are classified as “critical priority pathogens”, the highest category of the WHO’s Bacterial Priority Pathogens List, research in this field is highly necessary [20].

Hence, this study aims to provide data on the resistance rate and MIC distribution of DFX in Enterobacterales that are resistant to older fluoroquinolones in order to answer the following question: Is it useful or dispensable to perform subsequent DFX testing in CIP-resistant isolates?

## 2. Results

### 2.1. Sample and Isolate Characteristics

Samples originated from a variety of sample sites (Table 1), wound swabs being the most common specimen (*n* = 77; 60%). Isolates comprised 13 different species, with the majority (88%) belonging to the species *Escherichia coli*, *Proteus mirabilis*, and *Klebsiella pneumoniae*. All included species and their respective isolate numbers can be found in Table 2. All included isolates were found to be resistant to CIP in VITEK 2 analysis as well as BMD (Table 3). MICs ranged from 1 mg L^−1^ to ≥ 16 mg L^−1^. The vast majority were found to be highly resistant (median ≥ 16 mg L^−1^). Most isolates tested were susceptible to other oral antibiotics (*n* = 83; 64%): forty-five (35%) and 59 (46%) isolates over all species were susceptible to AMC and SXT, respectively (Table 4). Twenty-two isolates (17%) were identified as positive for extended spectrum beta-lactamases. Two isolates (both *K. pneumoniae*; 2% of all isolates) were carbapenem resistant, both of which tested positive for a carbapenemase: one OXA-48-like and one NDM, respectively.

### 2.2. Delafloxacin MICs

**Figure 1:**
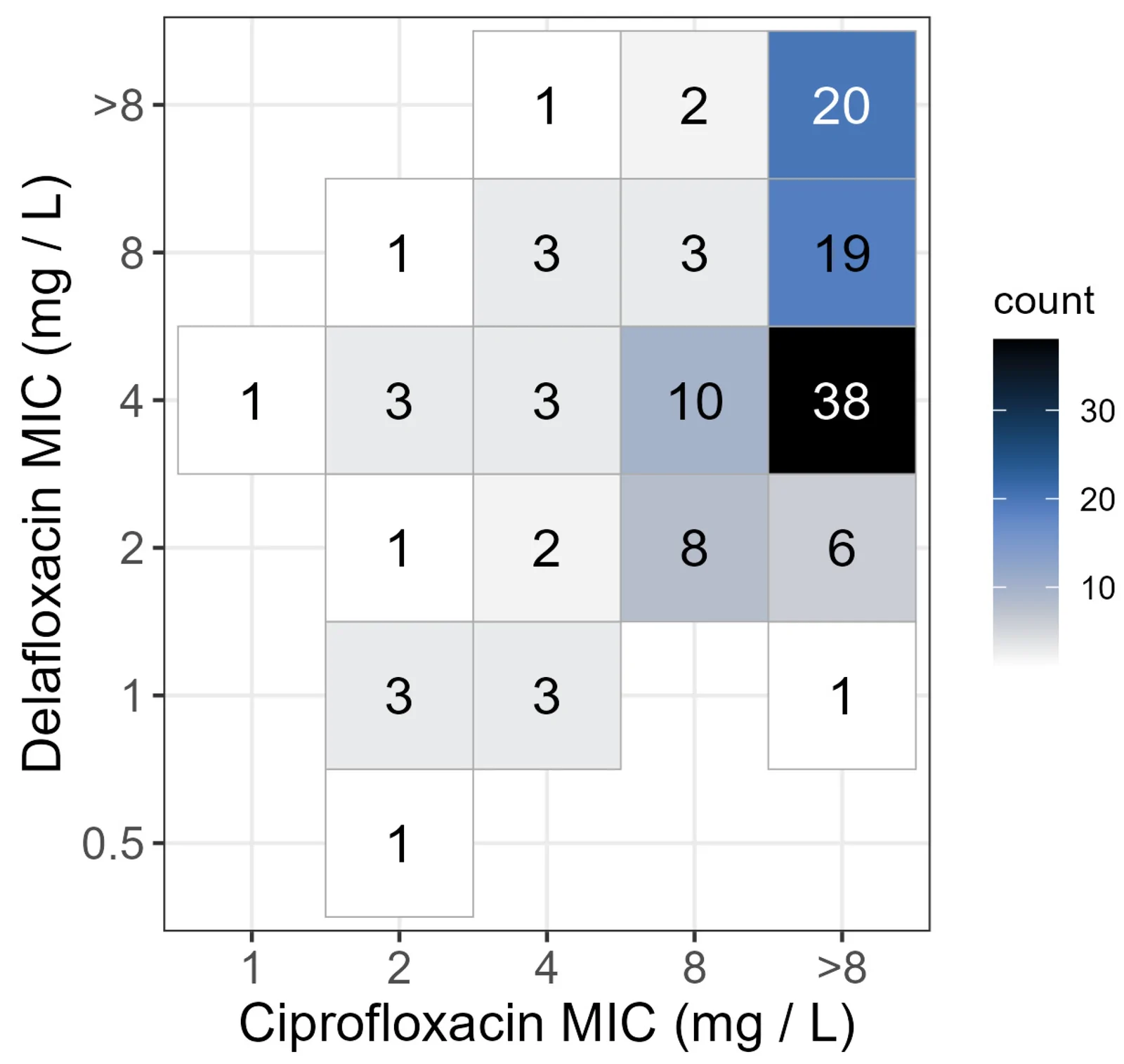
Heat map depicting the distribution of ciprofloxacin and delafloxacin MICs per isolate.

Over all 129 included isolates, a median MIC of 4 mg L^−1^ was measured, with MICs ranging from 0.5 to ≥ 16 mg L^−1^. Figure 1 shows the distribution of ciprofloxacin and delafloxacin MICs per isolate. Despite starting the microdilution assay at a concentration of 0.008 mg L^−1^, no low MIC values were detected.

EUCAST only defines clinical breakpoints for DFX susceptibility (MIC ≤ 0.125 mg L^−1^) and resistance (MIC > 0.125 mg L^−1^) for *E. coli*. None of the 56 *E. coli* isolates (representing 43% of all included isolates) were susceptible to DFX: with a median MIC of 4 mg L^−1^ and a minimum MIC of 0.5 mg L^−1^, all *E. coli* were clearly resistant. Only one *E. coli* reached an MIC of 0.5 mg L^−1^, placing itself within the confidence interval (CI) of EUCAST’s ECOFF values.

Although there are no specific breakpoints for other species, ECOFFs are available for 64 of the remaining 73 non-*E. coli* isolates (89%, representing 50% of all included isolates), most notably *Proteus mirabilis* (ECOFF 0.25; *n* = 45; 35%) [21]. For *Citrobacter koseri*, EUCAST currently only defined a tentative ECOFF. No isolate recorded an MIC below or at the (tentative) ECOFF. Again, the median was 4 mg L^−1^, with a minimum MIC of 1 mg L^−1^. The two isolates with a 1 mg L^−1^ MIC, thus clearly above the respective ECOFFs, belonged to the species *K. pneumoniae* and *P. mirabilis*. Applying the interpretative criteria for *Escherichia coli*, *Klebsiella pneumoniae*, and *Enterobacter cloacae* of Mogle et al., which were based on suggestions by the drug manufacturer, only the one mentioned *E. coli* isolate would have been interpreted as “I” [22]. All other isolates would still fall into the “R” category.

**Figure 2:**
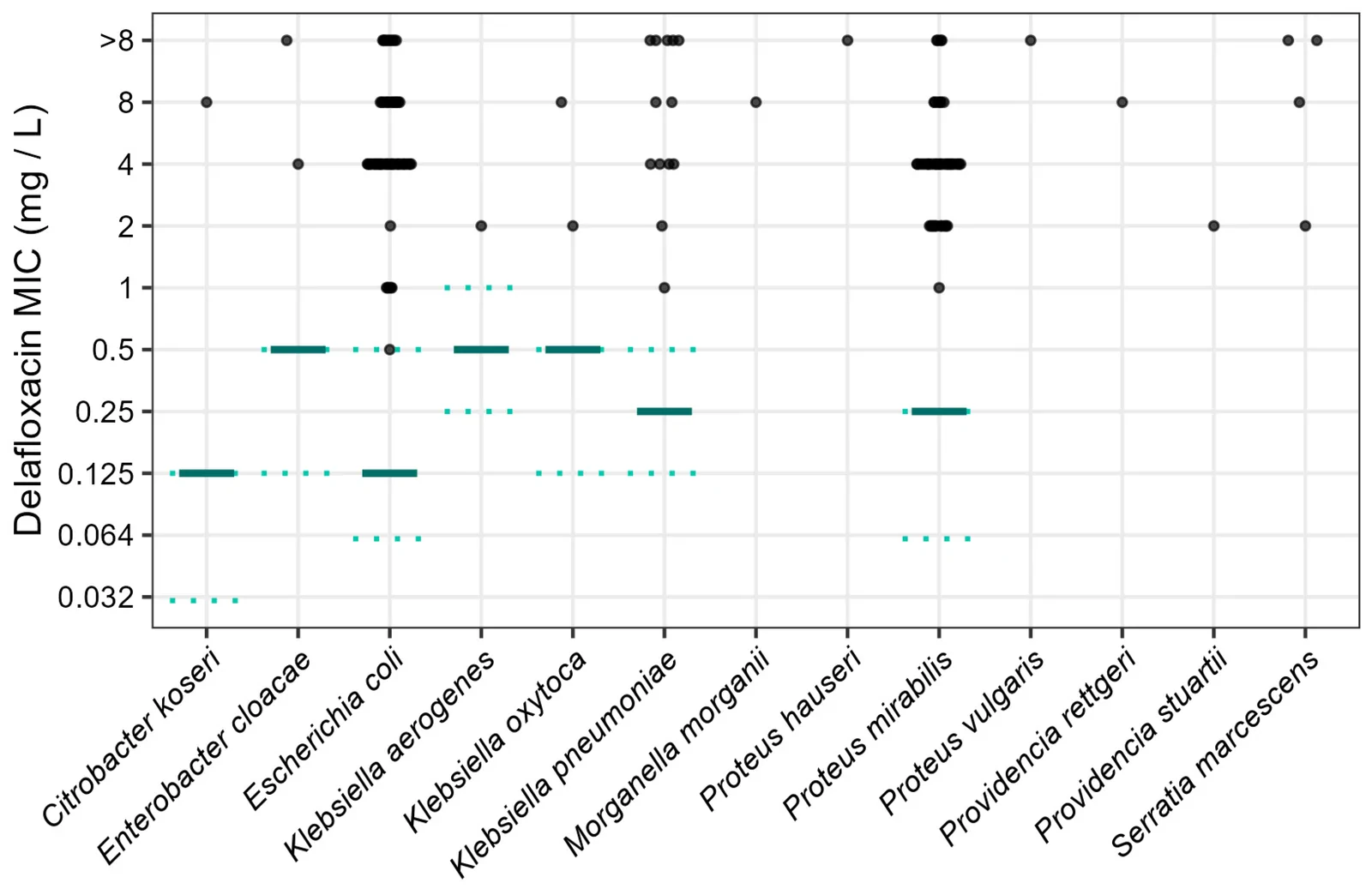
Distribution of minimum inhibitory concentrations (MICs) for delafloxacin for the included isolates. Each black dot represents one isolate. (Tentative) epidemiological cut-off ([T]ECOFF/ECOFF) values according to EUCAST are displayed in teal: solid lines indicate the (T)ECOFF/ECOFF values and dashed lines the respective 95% confidence interval. No reference lines are shown where no (T)ECOFF is available.

Eight included isolates (6%) belong to species for which EUCAST has neither published breakpoints nor ECOFFS: *Serratia marcescens* (*n* = 4), *Morganella morganii* (*n* = 1), *Proteus hauseri* (*n* = 1), *Providencia rettgeri* (*n* = 1), and *Providencia stuartii* (*n* = 1). With a median MIC of 8 mg L^−1^ and a minimum MIC of 2 mg L^−1^, these isolates, even though low in total number, tended to demonstrate even higher DFX MICs than the other strains included in this study (Figure 2).

Within the analyzed population, ciprofloxacin and delafloxacin MIC values showed a significant but weak positive ordinal correlation, calculated using a censored-date adjustment (Kendall’s tau-b = 0.21; *p* < 0.001; 95–CI 0.11–0.29). Exploratory species-specific estimates were calculated for species represented by at least 10 isolates. Kendall’s tau-b was 0.21 for *E. coli* (95–CI 0.08–0.33; *n* = 56), 0.47 for *K. pneumoniae* (95–CI 0.24–0.59; *n* = 13), and 0.16 for *P. mirabilis* (95–CI −0.02–0.28; *n* = 45).

## 3. Discussion

Fluoroquinolones exert their bactericidal effect via inhibition of topoisomerases, enzymes catalyzing DNA cleavage and resealing in order to interconvert relaxed and supercoiled forms of DNA [3]. In Gram-negative bacilli, the main fluoroquinolone target is gyrase, while it is topoisomerase IV in Gram-positive bacteria [3]. While most fluoroquinolones target primarily one type of enzyme, DFX is active against both [13,14]. Whilst resistance against most fluoroquinolones is acquired by a single-point mutation in a highly conserved region in one of the genes encoding subunits of the polymeric topoisomerases, e.g., *gyrA* or *parC* [6,7], DFX resistance relies on multiple-point mutations in both topoisomerases, which should theoretically slow down the development of resistance [3,7,15]. In this study, we found that all 129 included CIP-resistant Enterobacterales from clinical samples were also either resistant to DFX, or, in cases where no breakpoints are available, yielded MICs above the respective ECOFF. In the absence of species-specific breakpoints, this indicates non-wild-type isolates; however, it is not possible to classify them as resistant. When excluding a single *E. coli* that reached an MIC corresponding to the upper limit of the confidence interval, all isolates were placed clearly above that margin (Figure 2). In addition, our data also shows a positive correlation between the MIC values of the two fluoroquinolones. Currently, there is a range of publications available investigating DFX resistance rates in specific clinical settings, e.g., arthritis or keratitis [6,15], or specific bacterial genera, e.g., *Corynebacterium* or *Ureaplasma* [16,17]. However, for a major group, which is more common in clinical infectiology and medical microbiology, data is still lacking: Enterobacterales. Only a few publications focused on this group, and more than a decade has passed since [18,19]. Besides the fact that the epidemiology has presumably changed since then, the authors chose to also include isolates without preexisting resistance against lower-generation fluoroquinolones. In our view, however, this is a prerequisite for the use of a novel and more expensive fourth-generation drug, especially if knowledge and experience about adverse effects is still comparably limited [23]. Previous studies, albeit in different organisms, have reported increased susceptibility to DFX in isolates with resistance towards lower-generation fluoroquinolones. For instance, DFX was highly effective in vitro against levofloxacin-resistant pneumococci or staphylococci [6,24], and two recent studies detected DFX susceptibility in about one third of CIP-resistant *Pseudomonas aeruginosa* isolates (30% and 36%, respectively) [25,26]. The latter studies and a publication by Biagi et al., showing that DFX was the least active fluoroquinolone in *Stenotrophomonas maltophilia* [27], indicate that the findings in Gram-positive organisms cannot be easily conveyed to Gram-negative organisms. Our results underscore this verdict: Although MICs were lower for DFX (median of 4 mg L^−1^) compared to CIP (median of ≥16 mg L^−1^), they were clearly not low enough to render the isolates susceptible. Unfortunately, comparable data for these Gram-negative bacilli are limited.

While we directly compared DFX–MIC values to CIP–MIC values for each individual isolate, the previous studies—due to their differing intention—only allow a comparison of overall resistance rates. Some published results contain a small number of Enterobacterales tested for both CIP and DFX [13,28,29,30]. There, the reported susceptibility rates for the two drugs are comparable, indicating that DFX does not add significant value compared to CIP [13,28,29,30]. Additionally, for multidrug-resistant pathogens, DFX, which is often proclaimed as a reserve antibiotic, does not seem to provide a benefit over CIP, as evidenced by comparable resistance rates in ESBL-positive high-risk *E. coli* strains (ST43) for both substances [30]. The same accounts for comparisons to other second- and third-generation fluoroquinolones like levofloxacin and moxifloxacin [14,29,30]. The last to pose the same research question as we did were Pfaller et al., who, based on an isolate collection sampled in Europe and the US before 2014, observed that more than 90% of fluoroquinolone-resistant Enterobacteriaceae (but not Enterobacterales) displayed decreased susceptibility to DFX, which predicted a development resulting in the epidemiology that we report today [18].

Altogether, our findings suggest that DFX testing can be waived in CIP-resistant *E. coli* and might be of limited value in other species of Enterobacterales.

This study has some limitations that need to be considered. Firstly, the number of analyzed isolates is limited. However, it is unclear whether expanding the sample size would significantly decrease the obtained rates of DFX resistance or non-wild-type ECOFFs. In addition to that, no sequencing data is available for the included isolates, making it impossible to rule out that specific clonal lines might be overrepresented. Secondly, these rates were calculated from the results of a single center, which might not be representative of the epidemiology in other regions. Thirdly, this study included Enterobacterales isolated from a wide range of clinical specimens, whereas the antibiotic is currently only approved for the treatment of ABSSSI and CAP. For the same reason, we excluded all isolates originating from the urinary tract, and hence cannot present susceptibility data for urinary tract infections, which are a major indication for fluoroquinolone administration. While it is possible that our very clear result may not be transferable to those Enterobacterales isolates that cause non-ABSSSI and non-CAP infections, this can be considered highly unlikely.

## 4. Methods

This study was performed at the Diagnostic and Research Institute for Hygiene, Microbiology and Environmental Medicine in Graz, Austria, which provides microbiological diagnostics for the University Hospital of Graz, a 1600-bed tertiary care center, and more than 1600 medical practices in southeastern Austria. The isolate collection was performed prospectively over six months between February and July 2024 and included all non-duplicate isolates (maximum of one isolate per species per patient) of ciprofloxacin-resistant Enterobacterales, originating from clinical samples other than urine (*n* = 129). Species identification was carried out via MALDI-TOF MS (MALDI Biotyper sirius, Bruker Daltonics, Bremen, Germany). Before they were unthawed and incubated overnight at 35 ± 2 °C, isolates were stored at −80 °C. Growth of colonies was visually checked for purity by skilled laboratory personnel. All isolates were passaged on Columbia blood agar (BD Columbia Agar with 5% Sheep Blood, ref. 254071; Becton Dickinson, Franklin Lake, USA) prior to conducting broth microdilution (BMD).

A BMD assay was prepared using CIP (ref. 17850; Sigma-Aldrich, St. Louis, MO, USA) and DFX (ref. SML1869; Sigma-Aldrich) powder. All procedures were carried out according to EUCAST protocols and ISO 20776-1 [31,32]. The 1:1 dilution steps in un-supplemented cation adjusted MH-broth (Sensititre MH broth, ref. YT3462; TREK Diagnostic Systems Ltd., East Grinstead, UK) were performed starting from sterile-filtered 128 mg L^−1^ stock solutions of CIP in ddH_2_O and DFX in DMSO (ref. D4540; Merck, Darmstadt, Germany). Then, 50 µL of the antibiotic solutions were transferred into the wells of sterile round-bottom 96-well microtiter plates (Nunc 96-Well Polystyrene Round Bottom Microwell Plates, ref. 262162; Thermo Fisher Scientific, Waltham, MA, USA) before 50 µL of a 10^6^ CFU mL^−1^ bacterial suspension was added for the final concentration of 5 × 10^5^ CFU ml^−1^. The final range of minimum inhibitory concentrations (MICs) after addition of the bacterial suspension for both substances stretched from 0.008 to 8 mg L^−1^ (11 wells), with the last well containing a growth control for each isolate. The DFX growth control contained the same DMSO concentration as all DFX wells, i.e., 0.31%.

Results were read after incubation for 18–24 h at 35 ± 2 °C by two skilled microbiologists. In cases of disagreement, a third specialist was consulted. For quality control (QC) purposes, strain ATCC 25922 (*Escherichia coli*, ref. DSM 1103; DSMZ—German Collection of Microorganisms and Cell Cultures, Braunschweig, Germany) was used on each test day and yielded a result within the accepted QC range (0.008–0.03 mg L^−1^), i.e., an MIC of 0.008 mg L^−1^. QCs of non-filtered antibiotic showed no difference to filtered stock solutions.

Susceptibility tests for amoxicillin/clavulanic acid (AMC) and trimethoprim/sulfamethoxazole (SXT) were carried out using a VITEK 2 card AST-N433 (ref. 424389; bioMérieux, Marcy-l’Étoile, France) on a VITEK 2 XL device (bioMérieux).

Categorization of isolates as susceptible or resistant was inferred from EUCAST Breakpoint tables for interpretation of MICs and zone diameters, Version 15 [33]. Epidemiological cut-off values (ECOFF) were taken from the respective EUCAST website https://mic.eucast.org (accessed on 18 August 2026).

Data accumulation and analysis was performed using Microsoft Excel (Microsoft Corporation, Redmond, WA, USA) and R version 4.6.1 (R Foundation for Statistical Computing, Vienna, Austria.) Kendall’s Tau-b was calculated using the Akritas-Theil-Sen estimator implemented in cenken() (NADA2 package, version 2.0.2) to assess the ordinal correlation between ciprofloxacin and delafloxacin MIC values. Both variables were multiplied by −1 prior to analysis to correctly accommodate the right-censored MIC values, i.e., isolates with MIC > 8 mg L^−1^. The 95% confidence interval for Kendall’s Tau-b was calculated by bootstrapping (2000 replicates).

## 5. Conclusions

In conclusion, our results indicate that CIP resistance seems to be an excellent predictor of DFX resistance or non-wild-type MICs in Enterobacterales. Additionally, the majority of isolates tested in our cohort were susceptible to other oral antibiotics. Laboratories should investigate their local DFX resistance rates (for *E. coli*)/DFX MICs in CIP-resistant Enterobacterales in order to decide whether subsequent DFX susceptibility testing offers additional value.

## Figures and Tables

**Table 1:** Origin and number of the included isolates per sample site. LRT lower respiratory tract; URT upper respiratory tract.

	Isolates	
Sample Material	*N*	%
Total	129	100.0
Wound swab	77	59.7
Skin swab	14	10.9
LRT specimen	11	8.5
Stool (screening)	11	8.5
URT swab	5	3.9
Blood cultures	3	2.3
Drainage liquid/Punctation	3	2.3
Urethral swab	3	2.3
Tissue	2	1.6

**Table 2:** An overview of the included species, sorted by frequency.

	Isolates	
Organism	*N*	%
Enterobacterales	129	100.0
*Escherichia coli*	56	43.4
*Proteus mirabilis*	45	34.9
*Klebsiella pneumoniae*	13	10.1
*Serratia marcescens*	4	3.1
*Enterobacter cloacae* complex	2	1.6
*Klebsiella oxytoca*	2	1.6
*Citrobacter koseri*	1	0.8
*Klebsiella aerogenes*	1	0.8
*Morganella morganii*	1	0.8
*Proteus hauseri*	1	0.8
*Proteus vulgaris*	1	0.8
*Providencia stuartii*	1	0.8
*Providencia rettgeri*	1	0.8

**Table 3:** Results of delafloxacin broth microdilution in comparison to breakpoints (only available for *E. coli*: 0.125 mg L^−1^) and epidemiological cut-offs (ECOFFs) from EUCAST in mg L^−1^. The tentative ECOFF was used for *Citrobacter koseri*. Dashes indicate where no median minimum inhibitory concentration could be calculated and when no breakpoint or ECOFF was available.

Organism	Isolates (*n*)	Delafloxacin				
		Lowest MIC	Median MIC	*N* Susceptible (%)	ECOFF	*N* ≤ ECOFF (%)
Enterobacterales	129	0.5	4			0 (0.0)
*Escherichia coli*	56	0.5	4	0 (0.0)	0.125	0 (0.0)
*Proteus mirabilis*	45	1	4	-	0.25	0 (0.0)
*Klebsiella pneumoniae*	13	1	8	-	0.25	0 (0.0)
*Serratia marcescens*	4	2	>8	-	-	-
*Enterobacter cloacae* complex	2	4	8	-	0.5	0 (0.0)
*Klebsiella oxytoca*	2	2	4	-	0.5	0 (0.0)
*Citrobacter koseri*	1	8	-	-	0.125	0 (0.0)
*Klebsiella aerogenes*	1	2	-	-	0.5	0 (0.0)
*Morganella morganii*	1	8	-	-	-	-
*Proteus hauseri*	1	>8	-	-	-	-
*Proteus vulgaris*	1	>8	-	-	-	-
*Providencia stuartii*	1	2	-	-	-	-
*Providencia rettgeri*	1	8	-	-	-	-

**Table 4:** Susceptibility rates of all included isolates towards two other substances that are available for oral therapy. AMC, amoxicillin/clavulanic acid; SXT, trimethoprim/sulfamethoxazole.

Organism	Isolates (*n*)	Susceptible Isolates (*n*) and (%)	
		AMC	SXT
Enterobacterales	129	45 (34.9)	59 (45.7)
*Escherichia coli*	56	15 (26.8)	30 (53.6)
*Proteus mirabilis*	45	26 (57.8)	15 (33.3)
*Klebsiella pneumoniae*	13	2 (15.4)	6 (46.2)
*Serratia marcescens*	4	0 (0.0)	2 (50.0)
*Enterobacter cloacae* complex	2	0 (0.0)	1 (50.0)
*Klebsiella oxytoca*	2	1 (50.0)	2 (100.0)
*Citrobacter koseri*	1	0 (0.0)	1 (100.0)
*Klebsiella aerogenes*	1	0 (0.0)	1 (100.0)
*Morganella morganii*	1	0 (0.0)	0 (0.0)
*Proteus hauseri*	1	1 (100.0)	0 (0.0)
*Proteus vulgaris*	1	0 (0.0)	0 (0.0)
*Providencia stuartii*	1	0 (0.0)	1 (100.0)
*Providencia rettgeri*	1	0 (0.0)	0 (0.0)

## Data Availability

Original research data is available from the corresponding author upon reasonable request.
